# TLR9 agonism differentially impacts human NK cell-mediated direct killing and antibody-dependent cell-mediated cytotoxicity

**DOI:** 10.1038/s41598-024-65576-2

**Published:** 2024-06-25

**Authors:** Anna R. Mahr, Maia M. C. Bennett-Boehm, Frederik H. Rothemejer, Isabelle S. Weber, Alexander K. Regan, Josh Q. Franzen, Cami R. Bisson, Angela N. Truong, Rikke Olesen, Mariane H. Schleimann, Claudia M. Rauter, Audrey L. Smith, Dalia El-Gamal, Ole S. Søgaard, Martin Tolstrup, Paul W. Denton

**Affiliations:** 1 Department of Biology, University of Nebraska at Omaha, Omaha, NE USA; 2 Department of Interdisciplinary Informatics, University of Nebraska at Omaha, Omaha, NE USA; 3 Department of Infectious Diseases, Aarhus University Hospital, Aarhus, Denmark; 4 Department of Clinical Medicine, Aarhus University, Aarhus, Denmark; 5 Eppley Institute for Research in Cancer and Allied Diseases, University of Nebraska Medical Center, Omaha, NE USA; 6 Fred and Pamela Buffett Cancer Center, University of Nebraska Medical Center, Omaha, NE USA

**Keywords:** Human NK cells, Direct killing, ADCC, TLR9 agonist, Immunophenotyping, Immunotherapy, Innate immune cells, Innate immunity, Toll-like receptors

## Abstract

There are two known mechanisms by which natural killer (NK) cells recognize and kill diseased targets: (i) direct killing and (ii) antibody-dependent cell-mediated cytotoxicity (ADCC). We investigated an indirect NK cell activation strategy for the enhancement of human NK cell killing function. We did this by leveraging the fact that toll-like receptor 9 (TLR9) agonism within pools of human peripheral blood mononuclear cells (PBMCs) results in a robust interferon signaling cascade that leads to NK cell activation. After TLR9 agonist stimulation, NK cells were enriched and incorporated into assays to assess their ability to kill tumor cell line targets. Notably, differential impacts of TLR9 agonism were observed—direct killing was enhanced while ADCC was not increased. To ensure that the observed differential effects were not attributable to differences between human donors, we recapitulated the observation using our Natural Killer—Simultaneous ADCC and Direct Killing Assay (NK-SADKA) that controls for human-to-human differences. Next, we observed a treatment-induced decrease in NK cell surface CD16—known to be shed by NK cells post-activation. Given the essential role of CD16 in ADCC, such shedding could account for the observed differential impact of TLR9 agonism on NK cell-mediated killing capacity.

## Introduction

Natural killer (NK) cells are innate immune cells with the capacity to detect and kill diseased cells of various pathological origins. In humans, NK cells are defined by their surface levels of the canonical NK cell marker CD56 and lack of other lymphoid lineage markers including CD3 and CD19. Additionally, the presence or absence of activating and inhibitory surface markers determines the functionality of NK cells. Generally, cytokine-producing NK cells with regulatory capacity are CD56^bright^CD16^dim/neg^ while NK cells with cytotoxic capacity are CD56^dim^CD16^pos^^1^. NK cell maturation typically begins with a naïve, regulatory phenotype and progresses towards increased cytotoxic capacity, with some highly mature cells exhibiting a CD56^neg^CD16^bright^ surface phenotype^2^. Naïve regulatory NK cells also exhibit increased surface NKG2A levels corresponding to increased cytokine production and inhibition of cytotoxic capacities. As maturation progresses, CD56 and NKG2A are often downregulated while CD16, CD57, and killer Ig-like receptors (KIRs)—critical markers for the initiation and execution of cytotoxic functions—are upregulated^3^. Indeed, CD56^bright^ CD16^neg^ NK cells can be cytotoxic, although to a lesser extent than CD56^dim^ CD16^pos^ NK cells^3^. Cytotoxic NK cells can kill diseased cells by two distinct mechanisms, namely direct killing (mediated by non-MHC-I specific activating receptors, such as NCRs, DNAM-1 and NKG2D) and antibody dependent cell-mediated cytotoxicity (ADCC).

There are many important complexities and subtleties surrounding how NK cell activation/inhibition impact NK cell-mediated cytotoxicity. Activation/inhibitory signals are weighed against each other rather than being “on” or “off” switches that control killing functions. Despite these complexities, it can be helpful to consider the two killing approaches employed by NK cells in the context of basic ligand-ligand interactions. Direct killing is prompted when a target cell lacks major histocompatibility complex class I (MHC-I) on its surface, leading uninhibited receptors (e.g., KIRs, CD94/NKG2A, or LIR-1) on NK cells to initiate a missing-self signal. This lack of MHC-I/KIR interaction facilitates an activation signal mediated by activating receptors and ligands, thus triggering a cytotoxic response by the NK cell^4^. When a diseased target cell retains MHC-I on its surface, as is often the case in immune-evading cancers and cells infected by certain viruses, NK cells mediate ADCC. ADCC relies on the presence of a target-specific antibody, such as anti-CD20 (e.g., rituximab) in B cell lymphomas, to physically connect target and effector cells^5^. When the Fc region of the antibody is bound to an Fc receptor on the NK cell’s surface (e.g., FcγRIII, a.k.a. CD16a, referred to as CD16 hereafter) and the Fab region is bound to a target specific antigen (e.g., CD20), the target lymphoma B cell (in this example) is lysed.

NK cells are activated to carry out their cytotoxic functions through extracellular communication with other immune cells via cytokine signaling. Often, these communications are accomplished through the initiation of signaling cascades; one such cascade is triggered through toll-like receptor 9 (TLR9). Since NK cells typically only express TLR9 once they are activated^6^, TLR9 agonism-mediated impacts on NK cells are generally induced via a well-defined cell-to-cell signaling cascade that has been reviewed extensively (e.g.,^7–13^). In short, this cascade is potently initiated by CpG-rich DNA binding to intercellular TLR9 in plasmacytoid dendritic cells (pDCs), resulting in the release of type-I interferons such as interferon alpha (IFN-α)^13–15^. The type-I interferon response leads to a potent type-II interferon response by NK cells and other immune cells (e.g., macrophage). The resulting type-II interferon response creates a positive feedback loop between NK cells and macrophage through the production of interferon gamma (IFN-γ) and IP-10 respectively. This loop priming NK cells to perform effector functions against the detected threat^9,13^.

Immunotherapies, such as TLR9 agonists, are being investigated for their ability to activate the immune system to better respond to infections or malignancies. To this end, there are two classes of synthetic DNA molecules containing CpG motifs that function as TLR9 agonists: (i) oligodeoxynucleotides, called CpG-ODNs, and (ii) double stem loop immunomodulators (dSLIMs)^16^. CpG-ODNs are the canonical class of TLR9 agonists and are composed of linear pieces of single stranded DNA which require stabilization to avoid potential degradation by endonucleases which can lead to off-target effects^13,17–19^. Thus, dSLIM TLR9 agonists (such as lefitolimod) were developed. dSLIM molecules are comprised of two CpG-containing single stranded loops at the ends of a covalently closed double stranded stem. The closed loop structure of dSLIM molecules eliminates the requirement for extraneous stabilization, allowing for lower toxicity and higher tolerance by patients^13,18,20,21^. Within this context, we and others have boosted human immune responses with lefitolimod ex vivo [e.g.,^14,22^] and in vivo within clinical trials [e.g.,^20–27^]. Thus, it is well documented that lefitolimod is immunostimulatory in humans.

NK cells recognize antibodies through FcγRIII (i.e., CD16) and are the predominant ADCC effector cells^28^. TLR9 agonism activates NK cells and increases their ability to kill target cells (e.g., cells with low MHC-I levels) through direct killing^14^. These facts raise the following question: Does treatment with TLR9 agonist enhance the capacity of NK cells to mediate ADCC? A survey of the TLR9 agonist literature suggests “yes”^7,8,13^. Yet, the answer to the experimental question is not simple. The key literature produced on this topic predates the wide availability of quality strategies for cell isolation (e.g., microbead-based magnetic enrichments) and was generated using bulk cell cultures as effectors in killing assays. In some cases, the evaluation of ADCC was performed using mixed cell population (e.g., whole blood or PBMC pools) with an assumption that most ADCC would be NK cell mediated^29,30^. In other cases, there was an explicit recognition that the cell(s) responsible for ADCC could be from multiple lineages^31^. Thus, ADCC ascribed to NK cells^7,8^ could possibly have been mediated by any of the several other leukocyte lineages (e.g., granulocytes, monocytes, and γδ T cells) that can also mediate ADCC through various Fcγ receptors. Nevertheless, based on these data, multiple reports [including one of our own^24^] have suggested that TLR9 agonist-based interventions in multiple disease contexts can lead to TLR9 agonist-induced improvements in human NK cell-mediated ADCC^12,24,30,32^. Yet despite the large body of publications and extensive clinical development of TLR9 agonists as clinical interventions or vaccine adjuvants^33,34^, the primary data supporting a TLR9 agonist-related improvement of NK cell-mediated ADCC are very limited and two of the most prominent papers addressing this point directly worked with mice and not human cells^29,31^. The concept that TLR9 agonists induce NK cells to mediate ADCC is present in the literature, but there are no published data showing that human NK cells, in fact, exhibit an enhanced capacity to mediate ADCC following TLR9 agonism. Thus, we first set out to confirm that TLR9 agonism indeed increases human NK cell-mediated ADCC. Surprisingly, we found that lefitolimod differentially impacts human NK cells’ ability to mediate direct killing as compared to ADCC. Our immunophenotyping of NK cells during the experiments pointed to a mechanistic explanation to explain the surprising differential effect on human NK cell function that we observed.

## Results

### Lefitolimod showed differential impacts on NK cell-mediated killing

**Figure 1:**
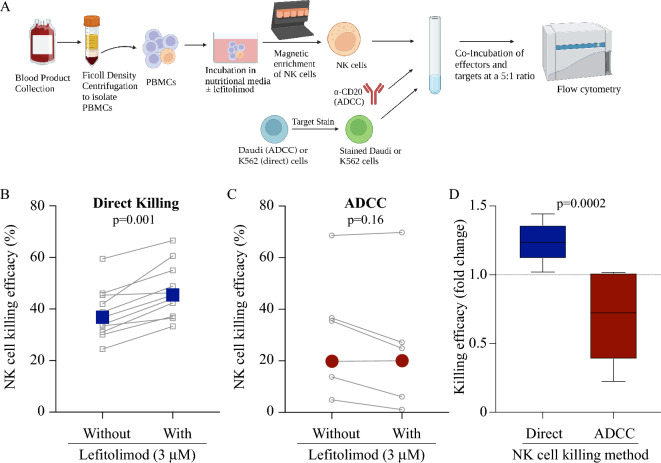
Lefitolimod causes differential effects on human NK cell-mediated killing strategies ex vivo*.* (**A**) Schematic detailing base NK cell-mediated killing assay methodological approach where K562 cells are the direct killing targets and Daudi cells are the ADCC targets. Figure generated using BioRender. (**B,C**) Human NK cell direct killing (**B**; n = 11) and ADCC (**C**; n = 6) outcomes ± lefitolimod graphed with grey symbols/lines corresponding to individual donors. Large colored data points (blue & red) represent the mean. (**D**) Box and whisker plots representing “with” fold change values from panels A and B in Supplemental Fig. 2 to substantiate the differential impact of lefitolimod treatment on direct killing versus ADCC. Statistics: Wilcoxon test used for panels (**B**–**D**).

First, we confirmed that our ex vivo incubation conditions resulted in the expected interferon signaling response^7–15^. To accomplish this, we evaluated changes in the levels of IFN-α2a, IFN-γ, and IP-10 as these cytokines are well-defined sentinels of the effects due to TLR9 agonism in human PBMCs^9,14,21,25^. We measured supernatants from PBMCs cultured for ~ 60 h ± lefitolimod (n = 11) (Supplemental Fig. 1). For IFN-α2a, the mean concentration for the unstimulated culture was 0.29 pg/mL (± 0.29 SEM) while the concentration in the stimulated culture was 22.8 pg/mL (± 9.9 SEM) (Wilcoxon test p value = 0.031). For IFN-γ, the mean concentration for the unstimulated culture was 158.5 pg/mL (± 28.6 SEM) while the concentration in the stimulated culture was 919.3 pg/mL (± 169.7 SEM) (Wilcoxon test p value = 0.001). For IP-10, the mean concentration for the unstimulated culture was 18,094 pg/mL (± 5103 SEM) while the concentration in the stimulated culture was 62,069 pg/mL (± 7048 SEM) (Wilcoxon test p value = 0.001). These increased levels of IFN-α2a, IFN-γ, and IP-10 indicate that lefitolimod activated human PBMCs in our experiments. To assess the functional implications of this activation, NK cell-mediated direct killing efficacy was assessed against K562 leukemia cells (Fig. 1A). Increased direct killing capacity was demonstrated by NK cells from lefitolimod-treated PBMCs when compared to NK cells from untreated PMBCs (n = 11) PBMC donors (Fig. 1B); which is in agreement with NK cell degranulation data we previously reported^14^. Despite the increased ability of human NK cells to mediate direct killing following lefitolimod treatment (Fig. 1B), NK-mediated ADCC was not enhanced as originally expected (Fig. 1C). These contrasting outcomes were highlighted when analyzed in the context of donor-specific fold changes. Specifically, all donors analyzed for direct killing efficacy showed positive fold changes (Supplemental Fig. 2A) while four of six donors analyzed for ADCC efficacy produced negative fold change values, indicating the inability of lefitolimod primed NK cells to enhance ADCC (Supplemental Fig. 2B). To clearly visualize the differential impact of lefitolimod on NK cell-mediated killing, “with” lefitolimod fold change values are shown for both direct killing (Supplemental Fig. 2A), ADCC (Supplemental Fig. 2B), and using box and whisker plots (Fig. 1D) the NK killing efficacy with lefitolimod fold change is displayed for both direct killing and ADCC. Based on these data, lefitolimod treatment boosts the ability of NK cell-mediated direct killing; however, this boost is not reflected in NK cell-mediated ADCC.

### Differential effects of lefitolimod on human NK cell killing function was independent of differences between donors

**Figure 2:**
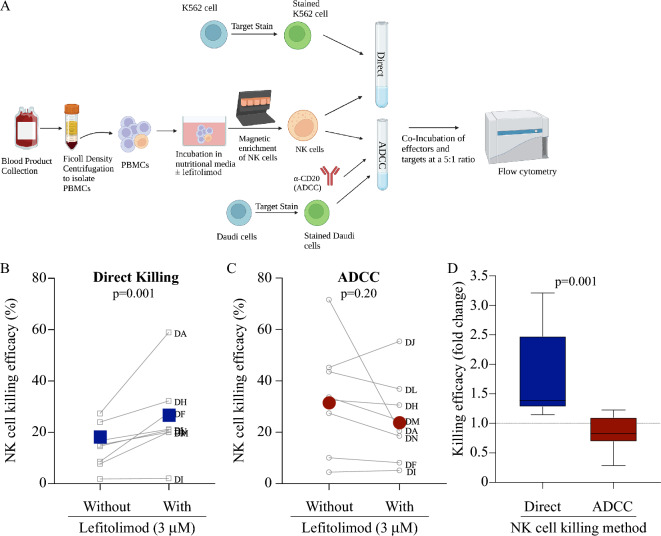
NK-SADKA controls for donor differences and the results confirm differential effects of lefitolimod on NK cell cytotoxic functions. (**A**) Schematic detailing the Natural Killer cell Simultaneous ADCC and Direct Killing Assay (NK-SADKA) methodological approach. Figure generated using BioRender. (**B,C**; n = 8) Human NK cell direct killing (**B**) and ADCC (**C**) outcomes ± lefitolimod graphed with grey symbols/lines corresponding to individual donors. Large colored data points (blue & red) represent the mean. (**D**) Box and whisker plots representing “with” fold change values from panels A and B of Supplemental Fig. 3 to substantiate the differential impact of lefitolimod treatment on direct killing versus ADCC. Each deidentified donor “D” assigned a new alphabetical identifier (e.g., DA for Donor A). Statistics: Wilcoxon test used for panels (**B**–**D**).

To assess whether the differential outcomes observed in Fig. 1 were attributable to differences between donors, we utilized our Natural Killer cell Simultaneous ADCC and Direct Killing Assay (NK-SADKA) method to quantitate both direct killing and ADCC efficacy simultaneously using NK cells from the same donors (Fig. 2A)^35^. Because cells from the same human donor are tested for both direct killing and ADCC within the NK-SADKA, an increase in direct killing in response to lefitolimod in this assay serves as a positive control for effective lefitolimod stimulation such that it is possible to accurately interpretate any lack of change in ADCC by cells from the same stimulation culture. Importantly, NK-SADKA data recapitulated the initial assay outcomes for both direct killing and ADCC (Figs. 1, 2). In the NK-SADKA, lefitolimod treatment again enhanced NK cell direct killing (Fig. 2B) but not ADCC (Fig. 2C). When analyzing fold change values, each donor had an increase in direct killing (Supplemental Fig. 3A) and six of eight donors had a decrease in ADCC efficacy with treatment (Supplemental Fig. 3B). As in Fig. 1D, “with” lefitolimod fold change values are shown for both direct killing and ADCC using box and whisker plots (Fig. 2D). Moreover, surface levels of CD107a tracked with NK-SADKA outcomes for direct killing (in agreement with our prior data^14^) and for ADCC (Supplemental Fig. 4). Therefore, in addition to highlighting the utility of an assay such as the NK-SADKA to control for donor variability, these data confirm the differential impacts of lefitolimod on the ability of NK cells to mediate direct killing and ADCC.

**Figure 3:**
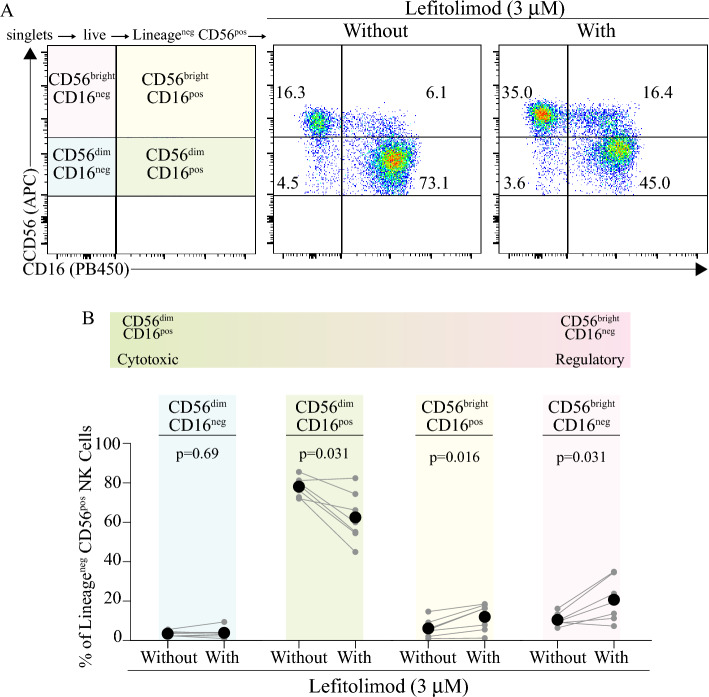
Lefitolimod treatment induces a shift from a cytotoxic phenotype to a regulatory phenotype in NK cells. (**A**) A diagrammatic plot illustrates the CD56/CD16 gating strategy (left). Representative flow data plots from a single human donor show NK cell phenotypes ± lefitolimod for ~ 60 h (center and right). NK cells were defined as Lineage^neg^ [CD3, CD14, CD19 (FITC)] CD56^pos^ (APC), then gated on the indicated CD56 (APC) and CD16 (PB450) phenotypes. (**B**) Levels of each NK cell CD56/CD16 phenotype are presented ± lefitolimod treatment (n = 7). NK cells were analyzed post 60-h PBMC co-stimulation and NK cell magnetic enrichment as depicted in (**A**). Statistics: Wilcoxon test used in panel (**B**).

### NK cell surface phenotype compositions were modulated in response to lefitolimod

To investigate the differential impacts seen between NK cell-mediated direct killing and ADCC, NK cell surface phenotypes were evaluated. The canonical phenotypes of NK cells ± lefitolimod were analyzed using surface levels of CD56 and CD16, two canonical markers of NK cell maturation and activation status (Table 1). We observed that NK cells isolated from PBMC pools that were treated with lefitolimod exhibited markedly differing CD56/CD16 surface phenotypes than those which had not been treated. To establish the exact phenotypic differences of NK cell populations with or without lefitolimod, four established NK cell subsets were assessed: CD56^dim^ CD16^neg^, CD56^dim^ CD16^pos^, CD56^bright^ CD16^pos^, and CD56^bright^ CD16^neg^ (Fig. 3A). Aggregated data from the assessed cohort showed that there was a significant decrease in the CD56^dim^ CD16^pos^ phenotype in treated NK cells when compared to non-treated NK cells across the 7 donors tested (Fig. 3B). There was also a significant increase in both CD56^bright^ CD16^neg^ and CD56^bright^ CD16^pos^ phenotypes in the treated NK cell samples.

**Table 1:** Reagents utilized for NK cell surface flow cytometric analyses.

Marker	Clone	Fluorophore	Purpose	Catalog
CD3	UCTH1	FITC	Lineage exclusion (T cells)	BioLegend #300406
CD14	HCD14	FITC	Lineage exclusion (macrophages, monocytes, granulocytes)	BioLegend #325604
CD19	HIB19	FITC	Lineage exclusion (B cells)	BioLegend #302206
CD56	HCD56	APC	Consensus NK cell marker	BioLegend #318310
CD16	3G8	Pacific Blue	NK cell marker, ADCC mediator	BioLegend #302032
CD107a	H4A3	PE-Cy7	Indicator of degranulation	Biolegend #328618
–	–	7-AAD	Live/dead discriminator	Stem Cell Tech #75001

### Surface CD16 levels decreased while soluble CD16 levels increased in response to lefitolimod

**Figure 4:**
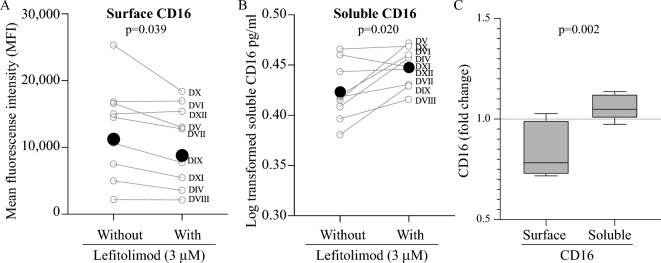
CD16 surface levels on NK cells treated with Lefitolimod is decreased while soluble levels of CD16 increase. (**A**) CD16 surface levels of Lineage^neg^CD56^pos^ cells evaluated via flow cytometry using our NK cell phenotyping panel (Table 1). Values represent CD16 surface levels ± lefitolimod treatment. At the time of analysis (post 60-h incubation) there was a decrease in the mean florescence intensity (MFI) of CD16 surface levels on NK cells exposed to lefitolimod. (**B**) ELISA analysis of bio-banked supernatants collected after 60-h incubation and cryopreserved until analysis detected significant increased soluble CD16 in samples exposed to lefitolimod as compared to those that were not. Paired data points connected with grey lines represent single human samples ± lefitolimod. Donors presented in panels A and B are identical. (**C**) Box and whisker plots representing treated “with” fold change values from panels A and B from Supplemental Fig. 5 to substantiate the surface and soluble CD16 levels. Each deidentified donor “D” assigned a new roman numeral identifier (e.g., DIV for Donor IV). Statistics: Wilcoxon test used for all panels.

After the surface phenotype of treated NK cells had been established (Fig. 3), we noted a significant decrease in surface CD16 levels (Fig. 4A). Given these findings and based on published data^36–38^, we hypothesized that lefitolimod agonism of TLR9 activates NK cells and subsequently causes CD16 to be shed from their surface prior to killing assay initiation. Culture supernatants from the same 9 donors in Fig. 4A were tested to determine whether CD16 was being shed from the surface of lefitolimod-treated PBMCs. Supernatants from the lefitolimod-treated PMBC cultures harbored significantly increased levels of soluble CD16 as compared to non-treated samples (Fig. 4B). While the source of the soluble CD16 could be NK cells or other cells, such as monocytes, in the PBMC pools, the data in Fig. 4A,B together point to reduced NK cell CD16 levels following treatment with lefitolimod. When analyzing fold change values, seven of nine donors had a decrease in surface CD16 (Supplemental Fig. 5A) and eight of nine donors had an increase in soluble CD16 with the treatment (Supplemental Fig. 5B). As in Figs. 1D and 2D, “with” lefitolimod fold change values are shown for both surface (Supplemental Fig. 5A) and soluble (Supplemental Fig. 5B) CD16 using box and whisker plots (Fig. 4C). These observations taken as a whole support our hypothesis that when PBMCs are exposed to lefitolimod, NK cells are primed for cytotoxic functions. But subsequently, the primed NK cells shed CD16 from their surface, reducing ADCC-antibody binding site availability. The outcome of this reduction is lower ADCC killing efficacy—even though the NK-SADKA demonstrated that the same pool of NK cells is primed to mediate increased direct killing.

## Discussion

**Figure 5:**
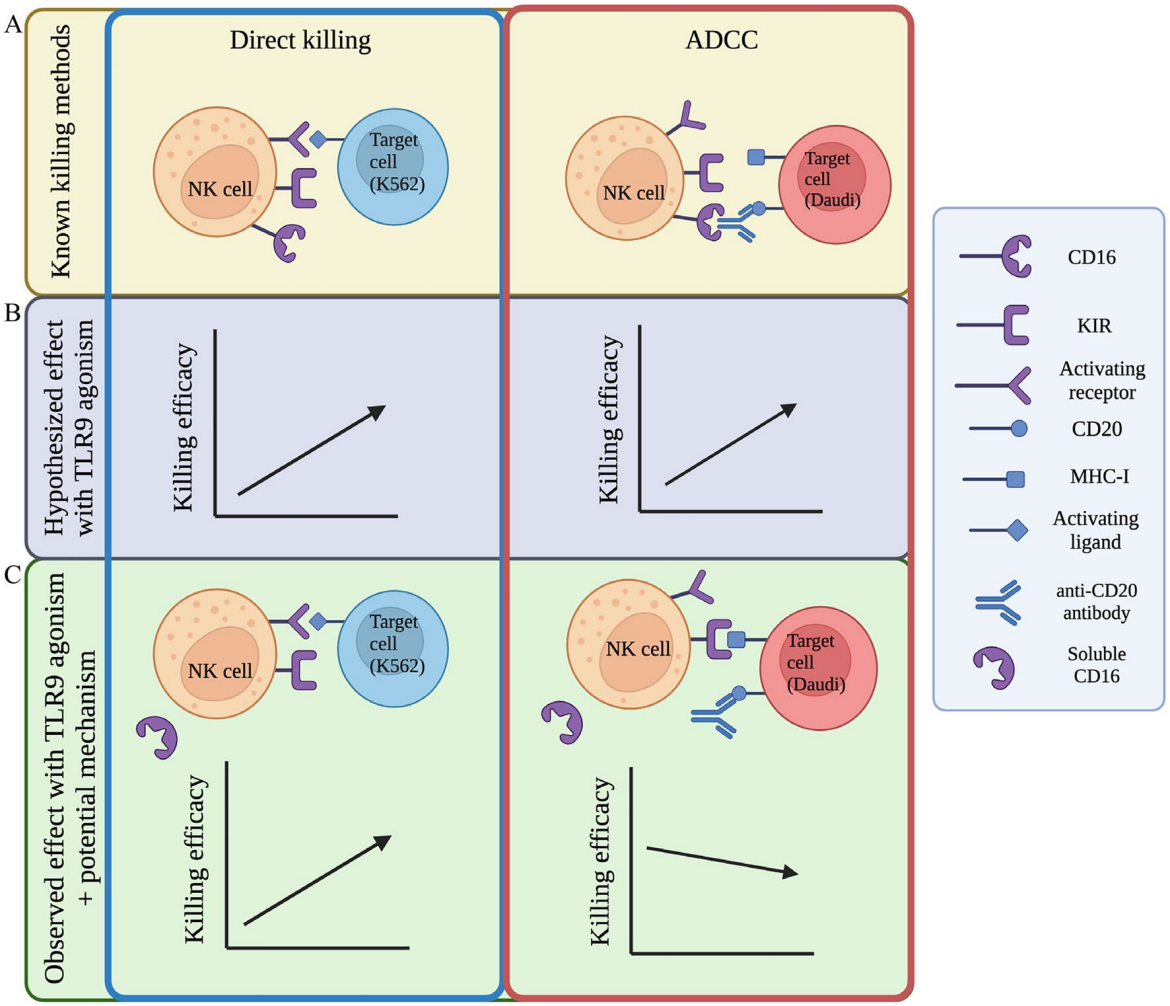
Surface CD16 levels do not dictate NK cells’ ability to mediate direct killing as with ADCC. (**A**) Mechanisms by which NK cells kill diseased cells. (**B**) Hypothesis tested: direct killing and ADCC are enhanced similarly with lefitolimod treatment. (**C**) The observed effect of lefitolimod on NK cell-mediated killing. Contents within vertical blue outline pertain to direct killing and contents within vertical red outline pertain to ADCC.

We set out to measure the effects of lefitolimod treatment on the ability of human NK cells to mediate their cytotoxic functions, namely direct killing and ADCC (Fig. 5A). Based on published literature surrounding the effect of TLR9 agonists on NK cell-mediated cytotoxicity^7,8,13,29–31^, we hypothesized that direct killing and ADCC would be affected similarly (i.e., both would be enhanced with lefitolimod treatment) (Fig. 5B). However, after a series of results (Fig. 1) contradicting existing literature^7,8,13,29–31^, we found it necessary to utilize our NK-SADKA that quantifies both methods of NK cell-mediated killing while controlling for donor differences. Analyses of data generated during this project revealed that lefitolimod has minimal impact on the ability of human NK cells to mediate ADCC while simultaneously improving NK cells’ ability to mediate direct killing (Figs. 1 and 2). The data also point to a lefitolimod-induced change (decrease) in surface CD16 levels on NK cells as a reason for the differential killing efficacy observed (Figs. 3, 4, and 5C).

Loss of surface CD16 could point to three possible responses by the NK cell: (i) the NK cells are becoming inactivated and CD16 is subsequently downregulated; (ii) the NK cells are activated, leading to the internalization of CD16; or (iii) the NK cells are activated, leading to shedding of CD16 from the cell surface during the incubation and before CD16 has the possibility to bind the anti-CD20 antibody within the context of the killing assay effector-target co-incubation. This third option is made plausible by published data indicating that NK cells’ predominant method of regulating CD16 levels is via shedding, unlike other CD16^pos^ cell lineages^39^. More specifically, there is a large body of literature that details how activation of human NK cells leads to a sheddase A disintegrin and metalloproteinase-17 (ADAM17)-mediated cleavage of CD16 from the surface of the NK cell (e.g.,^36–38^). Our ELISA data that revealed an increase in soluble CD16 in the culture supernatants of PBMCs treated with lefitolimod, together with published data^36–38^, support the third possible response. Future studies will confirm the role of ADAM17 in the cleavage of CD16 from NK cells in the context of TLR9 agonism-mediated activation. Notably, our experiments would have likely yielded a different outcome had they been performed using mouse cells, given that CD16 shedding does not occur on mouse neutrophils or NK cells^36,38^. Our proposed model (Fig. 5C) shows our conclusion that ADCC is not improved following lefitolimod stimulation due to TLR9 agonist-induced decreases in CD16 surface levels.

Key publications support the concept that NK cell surface phenotypes are directly linked to NK cell functions^1–3^. However, our data reveal an exception to this pattern. Similar to prior literature, we found that post-activation NK cells have decreased surface CD16 levels. We also saw a subsequent increase in CD56^bright^ cells. Specifically, we observed a marked shift of lefitolimod-treated human NK cell phenotypes from CD56^dim^ CD16^pos^ cells (traditionally considered activated and cytotoxic) to CD56^bright^ CD16^neg^ cells (traditionally considered cytokine-producing and non-cytotoxic). Contrary to expectations, this phenotypic shift (albeit measured with only two surface markers) still allowed for enhanced NK cell-mediated direct killing to occur, but only in the context of direct killing. The marginal impacts of lefitolimod on NK cell ADCC are interpreted to be due to the critical nature of CD16 as the Fcγ receptor that binds to the ADCC antibody used (anti-CD20) and not to the loss of killing capacity since direct killing efficacy still increased in the NK-SADKA. In the future, the utilization of additional surface markers could refine this analysis approach. However, it remains notable that changes in human NK cell surface phenotypes, even a two-marker phenotype, asymmetrically predicted functional assay outcomes, as NK cell populations which shifted to a predominantly CD56^bright^ CD16^neg^ “canonically regulatory” phenotype still exhibited increased direct killing capacity. Thus, the change (or lack of change) in ADCC function associated with lefitolimod treatment is not analogous to a change in overall cytotoxic potential of donor NK cells such that NK cell surface phenotypes should perhaps not be used as sole predictors of NK cell killing capacity.

In conclusion, TLR9 agonism asymmetrically impacts NK cell killing efficacies. Specifically, direct killing efficacy was enhanced with lefitolimod stimulation while ADCC efficacy largely remained static. This lack of increase in ADCC efficacy following lefitolimod treatment was not due to a loss in overall cytotoxic capacity but was instead due to CD16 shedding.

## Methods

### Blood product procurement and PBMC preparation

Deidentified blood products (i.e., buffy coats) from human donors were procured by the American Red Cross (Washington DC, USA) and shipped to the University of Nebraska at Omaha on ice. PBMCs were purified using Ficoll-Paque density gradient centrifugation (Beckman Coulter; 470×g, 25 min, 20 °C, 5/10 acceleration, 1/10 deceleration) then resuspended in RPMI 1640 medium (ATCC, cat# 30-2001) supplemented with 10% fetal bovine serum (FBS) (Avantor, cat# 97068-065) and 1% penicillin/streptomycin (VWR, cat# 21J195302) (cRPMI-10) at 10 × 10^6^ cells/mL. Cells were then aliquoted into six-well plates (Sarstedt, cat# 83.3920) at 5 × 10^6^/mL in 5 mL per well. Lefitolimod was added to experimental plates at a final concentration of 3 μM and all plates incubated at 37 °C with 5% CO_2_ for ~ 60 h.

### Target cells

Human Burkitt’s lymphoma (Daudi) cells (CCL-213, ATCC; ADCC target cells) were cultured in RPMI 1640 (ATCC, cat# 30–2001) supplemented with 20% FBS and 1% penicillin/streptomycin (cRPMI-20). Human chronic myelogenous leukemia (K562) cells (CCL-243, ATCC; direct killing target cells) were cultured in IMDM (Corning, cat# 10-016-CV) supplemented with 10% FBS and 1% penicillin/streptomycin (cIMDM-10). Both cell lines were incubated at 37 °C with 5% CO_2_. Daudi and K562 cells were passaged approximately every 48 h. Each passage was accomplished by reseeding 3 × 10^6^ cells (Daudi) and 1 × 10^6^ cells (K562) in 9 mL of their respective media plus 1 mL of conditioned media that was retained from the previous passage. Cells were used as target cells in killing assays between 5 and ~ 25 passages.

### NK cell magnetic enrichment

Human NK cells were enriched from PBMC cultures treated with or without lefitolimod using a Human NK Cell Isolation Kit (Miltenyi Biotec, cat #130-092-657) according to the manufacturer’s instructions. Typical purity of NK cells was 90% ± 3.8 (n = 9) based on percentage of Lineage^neg^ (CD3, CD14, CD19) and CD56^pos^ cells post-magnetic isolation.

### Target cell staining for effector-target discrimination

Daudi and K562 cells were resuspended separately in PBS and incubated with 1.2 μM 5(6)-carboxyfluorescein diacetate n-hydrosuccinimide ester (CFDA-SE; cat# 75003, Stemcell Technologies) for 3 min at 37 °C with a 1:1 (cell volume: stain volume) ratio. Cell staining was quenched via the addition of 2 mL RPMI 1640 medium without phenol red (Quality Biological, cat# 112-040-101) supplemented with 10% fetal bovine serum (FBS) (Avantor, cat# 97068-065) and 1% penicillin/streptomycin (VWR, cat# 21J195302) (cRPMI-NPR) followed by an incubation for 10 min at 37 °C and 5% CO_2_. Samples were centrifuged (350xg, 15 min, 20 °C), supernatant aspirated, and pellet resuspended in 10 mL PBS. Target cells were then centrifuged a second time (350×g, 15 min, 20 °C) to remove all residual CFDA-SE. Supernatants were aspirated, and cells were resuspended at a concentration of 2 × 10^5^ cells/mL in cRPMI-NPR.

### Co-incubation of assay components

Previously enriched NK cells and stained target cells were aliquoted into autoclaved 5 mL round-bottom polypropylene test tubes (Fisher Scientific, cat# 14-959-1B) at a 5:1 NK to target cell ratio. The anti-CD20 antibody, (rituximab; Selleck Chemical, #A20095MG) was added to ADCC samples as 4 μL per 1 mL cell (i.e., 0.05 mg/mL) suspension. Samples were covered loosely with aluminum foil and incubated at 37 °C with 5% CO_2_ for 2 h.

### Cell staining for flow cytometric analyses

Post 2-h incubation, samples were centrifuged (350×g, 15 min, 20 °C), the supernatant was decanted, and the cells were vortexed briefly to disrupt the pellet. For killing assessment, 10 μL of 7-aminoactinomycin D (7-AAD) (Stemcell, cat #75001) was added to the samples in the approximately 100 μL of residual supernatant, vortexed gently to mix, and samples were incubated for 10 min at 20 °C in the dark. For immunophenotyping, samples were centrifuged (350xg, 15 min, 20 °C) following the 2-h incubation. Then, the supernatants were decanted, and cells were vortexed briefly to disrupt the pellet. Samples were then blocked with human FcX block (BioLegend, cat#422302; 10 min). Following this, samples were stained (15 min) with a Lineage cocktail (anti-CD3, anti-CD14, anti-CD19), anti-CD56, anti-CD16, and/or anti-CD107a (Table 1). PBS was added to sample tubes to reach a final volume of 3 mL for centrifugation. Samples were centrifuged (350×g, 15 min, 20 °C), the supernatant decanted, and cells were vortexed briefly to disrupt the pellet. 5 μL 7-AAD were then added to the samples in the approximately 100 μL of residual supernatant. The cells were vortexed to mix and incubated for 10 min at 20 °C in the dark.

### Flow cytometric analyses

Flow cytometry data were collected using a Beckman Coulter CytoFLEX flow cytometer and analyzed with FlowJo v10.8.2 (BD). For killing determination, target cell death was established as the percentage of 7-AAD^pos^ cells in the total CFSE^pos^ population (stained target cells). NK cell killing efficacy for ADCC and direct assays was determined by the general formula:

$$ killing \, efficacy \, = \, \% \, target \, cell \, death \, \left( {experimental} \right) \, - \, \% \, target \, cell \, death \, \left( {control} \right). $$

Control for direct killing was % of stained dead K562 cells without NK cells present. Control for ADCC was % stained dead Daudi with NK cells without anti-CD20 antibody. For immunophenotyping (Table 1), Lineage^pos^ and 7AAD^pos^ cells were excluded. NK cells were established as Lineage^neg^CD56^pos^. NK cells were then evaluated for levels of surface CD16.

### Quantification of soluble IFN-α2a, IFN-γ, and IP-10

At 24, 48 and 65 h respectively, PBMC supernatants from designated wells were collected and cryopreserved in aliquots for later electrochemiluminescence cytokine analysis. Aliquots from all donors and all time points were thawed and diluted 1:2 in cRPMI. A single-plex kit was used for IFN-α2a analysis (Meso scale discovery (MSD), cat #K151ACB), a single-plex kit for IP-10 analysis (MSD, cat #K151NVD) and a multiplex kit that included IFN-γ analysis (MSD, cat #K15049D). A dilution series of the supplied calibrator stock was prepared for generation of a standard curve. Samples and calibrator dilutions were then added to the three supplied plates and incubated for 2 h. The wells were then washed, and the supplied detection antibody was added. This was incubated for 2 h, followed by washing and addition of reading buffer. Samples were then immediately analyzed on a Meso Scale QuickPlex SQ120 reader.

### Quantification of soluble CD16

Soluble CD16 in the supernatants from treated PBMC cultures were quantified using an enzyme-linked immunosorbent assay (ELISA) (sensitivity, 1.35 ng/mL: Invitrogen, EH181RB) according to the manufacturer’s instructions. In brief, frozen supernatants were first thawed at room temperature. These undiluted supernatants and human CD16 standards were pipetted in triplicate into the wells of a pre-coated 96-well CD16 ELISA plate. The standards of human CD16 had concentrations ranging from 0 to 320 ng/mL. TMB peroxidase system consisted of streptavidin–horseradish peroxidase, TMB substrate, and the manufacturer’s HCl Stop Solution. Following the ELISA, absorbances of the solutions were read at 450 nm using a BioTek Synergy LX plate reader. To determine the concentration of soluble CD16 in the experimental samples, the absorbances of the experimental samples were interpolated from the standard curve.

### Statistics

All statistical analyses (α = 0.05) were performed on Prism version 9.5.0 for macIOS, GraphPad Software, La Jolla, CA, USA. (www.graphpad.com). Wilcoxon signed-rank test was used to determine difference between treatment conditions (± lefitolimod) per NK cell-mediated killing method. Wilcoxon signed-rank tests also were used to determine difference in direct killing and ADCC with lefitolimod treatment. Fold change values for NK cell-mediated killing were calculated using the number of dead cells ± lefitolimod using the equation.

$$ Fold \, change \, = deadwith \, lefitolimod \, /dead \, without \, lefitolimod $$

### Supplementary Information

Supplementary Information.

## Data Availability

The data that support the findings of this study are available from the corresponding author upon reasonable request.

## References

[1] Poznanski SM, Ashkar AA (2019). What defines NK cell functional fate: Phenotype or metabolism?. Front. Immunol..

[2] Abel AM, Yang C, Thakar MS, Malarkannan S (2018). Natural killer cells: Development, maturation, and clinical utilization. Front. Immunol..

[3] Béziat V (2011). CD56brightCD16+ NK cells: A functional intermediate stage of NK cell differentiation. J. Immunol..

[4] Sentman CL, Olsson MY, Kärre K (1995). Missing self recognition by natural killer cells in MHC class I transgenic mice. A 'receptor calibration' model for how effector cells adapt to self. Semin. Immunol..

[5] Iannello A, Ahmad A (2005). Role of antibody-dependent cell-mediated cytotoxicity in the efficacy of therapeutic anti-cancer monoclonal antibodies. Cancer Metastasis Rev..

[6] Sivori S (2004). CpG and double-stranded RNA trigger human NK cells by Toll-like receptors: Induction of cytokine release and cytotoxicity against tumors and dendritic cells. Proc. Natl. Acad. Sci. USA.

[7] Krieg AM (2004). Antitumor applications of stimulating toll-like receptor 9 with CpG oligodeoxynucleotides. Curr. Oncol. Rep..

[8] Krieg AM (2006). Therapeutic potential of Toll-like receptor 9 activation. Nat. Rev. Drug Discov..

[9] Schmoll HJ (2014). Maintenance treatment with the immunomodulator MGN1703, a Toll-like receptor 9 (TLR9) agonist, in patients with metastatic colorectal carcinoma and disease control after chemotherapy: A randomised, double-blind, placebo-controlled trial. J. Cancer Res. Clin. Oncol..

[10] Colonna M, Trinchieri G, Liu YJ (2004). Plasmacytoid dendritic cells in immunity. Nat. Immunol..

[11] Hvilsom CT, Søgaard OS (2021). TLR-agonist mediated enhancement of antibody-dependent effector functions as strategy for an HIV-1 cure. Front. Immunol..

[12] Martinsen JT, Gunst JD, Højen JF, Tolstrup M, Søgaard OS (2020). The use of toll-like receptor agonists in HIV-1 cure strategies. Front. Immunol..

[13] Wittig B, Schmidt M, Scheithauer W, Schmoll HJ (2015). MGN1703, an immunomodulator and toll-like receptor 9 (TLR-9) agonist: From bench to bedside. Crit. Rev. Oncol. Hematol..

[14] Offersen R (2016). A novel Toll-like receptor-9 agonist, MGN1703, enhances HIV-1 transcription and NK cell-mediated inhibition of HIV-1 infected autologous CD4+ T-cells. J. Virol..

[15] Sivori S, Carlomagno S, Moretta L, Moretta A (2006). Comparison of different CpG oligodeoxynucleotide classes for their capability to stimulate human NK cells. Eur. J. Immunol..

[16] Schmidt M (2006). Cytokine and Ig-production by CG-containing sequences with phosphorodiester backbone and dumbbell-shape. Allergy.

[17] Kapp K, Kleuss C, Schroff M, Wittig B (2014). Genuine immunomodulation with dSLIM. Mol. Therapy. Nucleic Acids.

[18] Schmidt M (2015). Design and structural requirements of the potent and safe TLR-9 agonistic immunomodulator MGN1703. Nucleic Acid. Ther..

[19] Adamus T, Kortylewski M (2018). The revival of CpG oligonucleotide-based cancer immunotherapies. Contemp. Oncol. (Pozn.).

[20] Thomas M (2018). Immunotherapeutic maintenance treatment with toll-like receptor 9 agonist lefitolimod in patients with extensive-stage small-cell lung cancer: Results from the exploratory, controlled, randomized, international phase II IMPULSE study. Ann. Oncol..

[21] Vibholm, L. K. *et al.* Effects of 24 week toll-like receptor 9 agonist treatment in HIV-1+ individuals: A single-arm, phase 1B/2A trial. *AIDS (London, England)* (2019). 10.1097/QAD.0000000000002213 30932955

[22] Lende SSF (2022). CD169 (siglec-1) as a robust human cell biomarker of toll-like receptor 9 agonist immunotherapy. Front. Cell Infect. Microbiol..

[23] Krarup AR (2017). The TLR9 agonist MGN1703 triggers a potent type I interferon response in the sigmoid colon. Mucosal Immunol..

[24] Schleimann MH (2019). TLR9 agonist MGN1703 enhances B cell differentiation and function in lymph nodes. EBioMedicine.

[25] Vibholm L (2017). Short-course toll-like receptor 9 agonist treatment impacts innate immunity and plasma viremia in individuals with human immunodeficiency virus infection. Clin. Infect. Dis..

[26] Kapp K (2016). Distinct immunological activation profiles of dSLIM® and ProMune® depend on their different structural context. Immun. Inflamm. Dis..

[27] Kapp K (2019). Beneficial modulation of the tumor microenvironment and generation of anti-tumor responses by TLR9 agonist lefitolimod alone and in combination with checkpoint inhibitors. Oncoimmunology.

[28] Lo Nigro C (2019). NK-mediated antibody-dependent cell-mediated cytotoxicity in solid tumors: Biological evidence and clinical perspectives. Ann. Transl. Med..

[29] Wooldridge JE, Ballas Z, Krieg AM, Weiner GJ (1997). Immunostimulatory oligodeoxynucleotides containing CpG motifs enhance the efficacy of monoclonal antibody therapy of lymphoma. Blood.

[30] Friedberg JW (2009). Phase II study of a TLR-9 agonist (1018 ISS) with rituximab in patients with relapsed or refractory follicular lymphoma. Br. J. Haematol..

[31] van Ojik HH (2003). CpG-A and B oligodeoxynucleotides enhance the efficacy of antibody therapy by activating different effector cell populations. Cancer Res..

[32] Cheng Y (2020). In situ immunization of a TLR9 agonist virus-like particle enhances anti-PD1 therapy. J. Immunother. Cancer..

[33] Karapetyan L, Luke JJ, Davar D (2020). Toll-like receptor 9 agonists in cancer. Onco. Targets Ther..

[34] Sogaard OS (2010). Improving the immunogenicity of pneumococcal conjugate vaccine in HIV-infected adults with a Toll-like receptor 9 agonist adjuvant: a randomized, controlled trial. Clin. Infect. Diseases.

[35] Bennett-Boehm, M. M. C. *et al.* Development and implementation of natural killer cell simultaneous ADCC and direct killing assay. *Heliyon* In Press (2023). 10.1016/j.heliyon.2023.e22991 PMC10731071 38125417

[36] Jing Y (2015). Identification of an ADAM17 cleavage region in human CD16 (FcγRIII) and the engineering of a non-cleavable version of the receptor in NK cells. PloS one.

[37] Srpan K (2018). Shedding of CD16 disassembles the NK cell immune synapse and boosts serial engagement of target cells. J. Cell Biol.

[38] Wang Y (1833). ADAM17 cleaves CD16b (FcγRIIIb) in human neutrophils. Biochim. Biophys. Acta.

[39] Peruzzi G (2013). Membrane-type 6 matrix metalloproteinase regulates the activation-induced downmodulation of CD16 in human primary NK cells. J. Immunol..

