# Supplementary material for: TLR9 agonism differentially impacts human NK cell-mediated direct killing and antibody-dependent cell-mediated cytotoxicity
Source: Sci Rep. 2024 Jun 25;14:14595. doi: 10.1038/s41598-024-65576-2 (PMC11199698; doi:10.1038/s41598-024-65576-2)
Supplement: Supplementary file 1 — Supplementary Information. [file 41598_2024_65576_MOESM1_ESM.pdf]

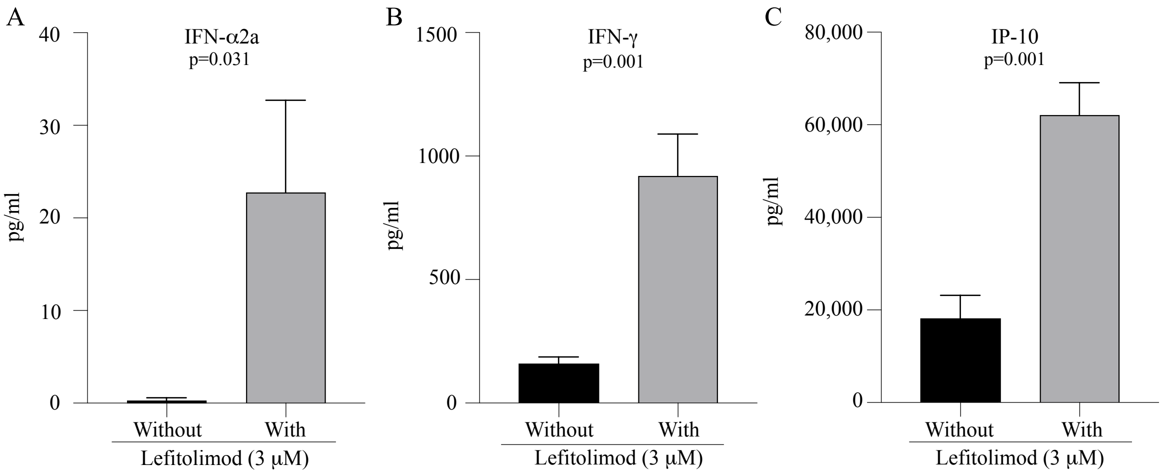

Supplemental Figure 1. Lefitolimod induces potent cytokine production *ex vivo*. (A-C) Concentrations of IFN-γ (A), IFN-α (B), and IP-10 (C) as measured in supernatants from PBMCs cultured for ~60 h ± lefitolimod (n=11). Data are presented as mean ± SEM. Statistics: Wilcoxon test.

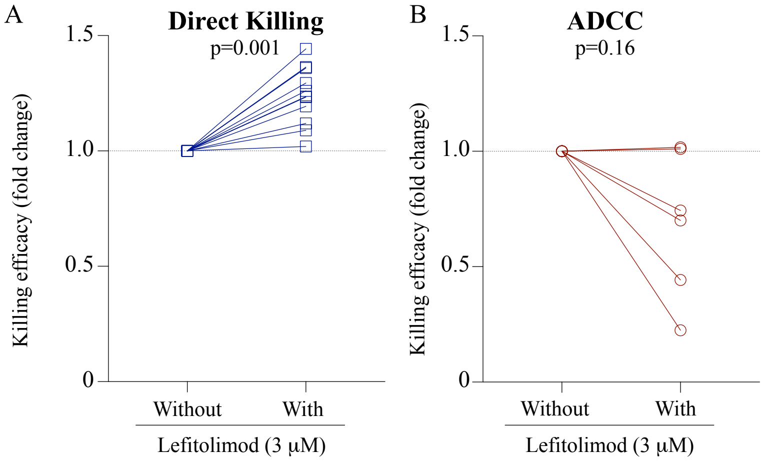

Supplemental Figure 2. Fold change data supporting Figure 1. (A-B) Fold change values highlight the differential impacts of lefitolimod on human NK cell direct killing (A) and ADCC (B). Statistics: Wilcoxon test.

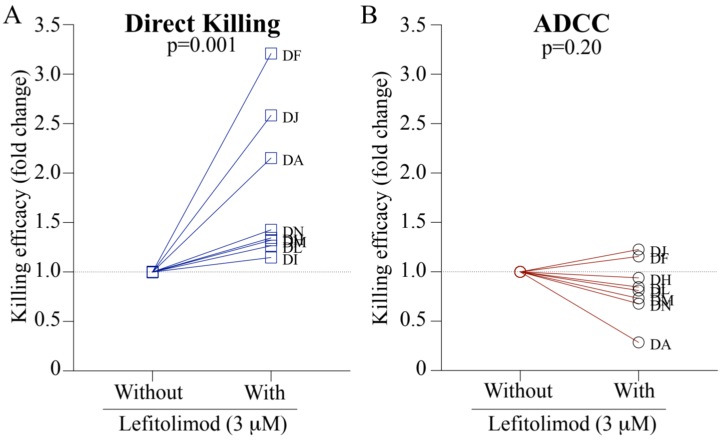

Supplemental Figure 3. Fold change data supporting Figure 2. (A-B) Fold change values highlight the differential impacts of lefitolimod on human NK cell direct killing (A) and ADCC (B). Statistics: Wilcoxon test.

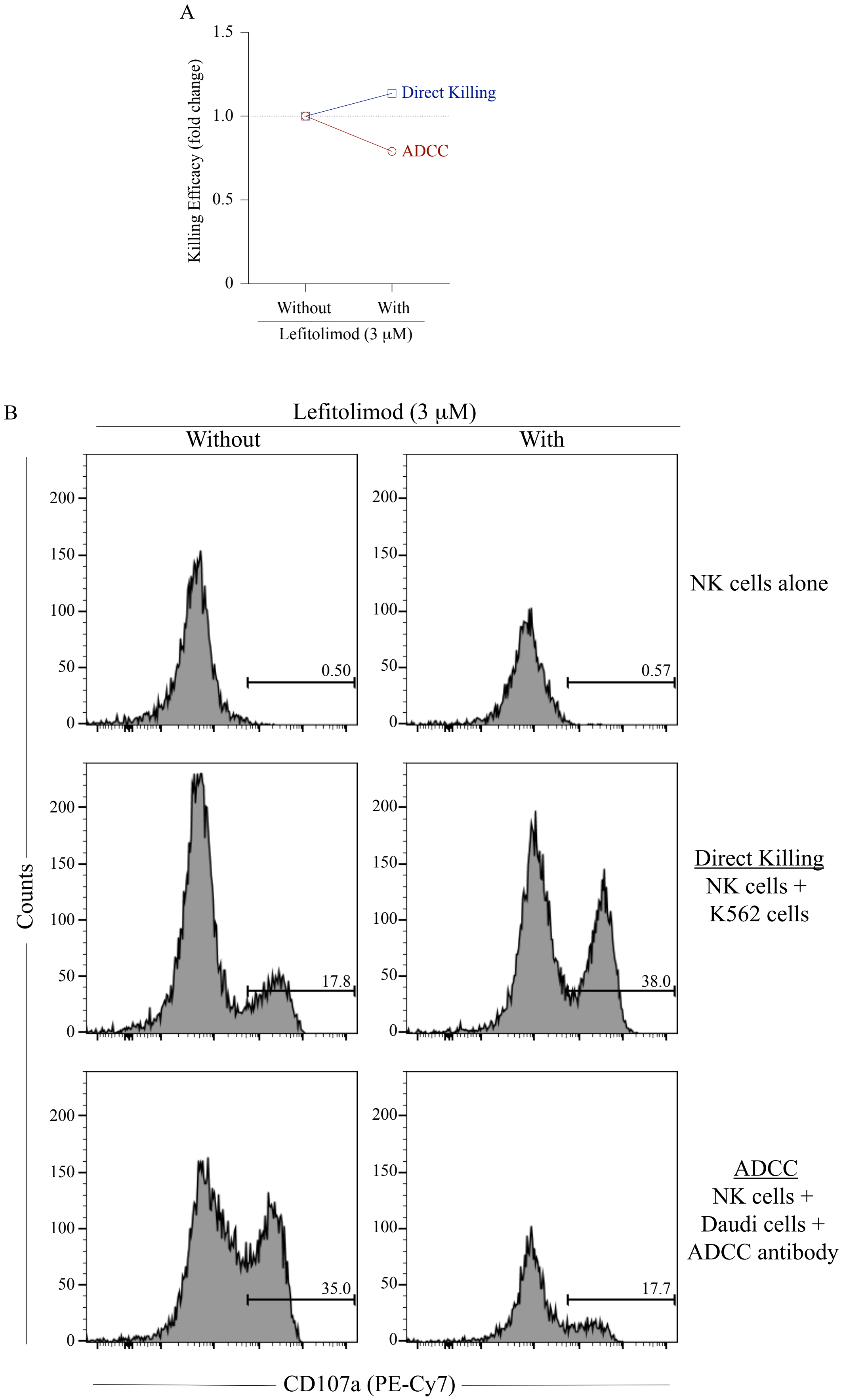

Supplemental Figure 4. CD107a degranulation patterns track with NK cell killing outcomes. (A) NK-SADKA outcomes show NK cell-mediated killing efficacy fold change shown for one human donor (representative of two tested). (B) Histograms showing CD107a levels on NK cells from the human shown in (A). The conditions evaluated are shown on the axes.

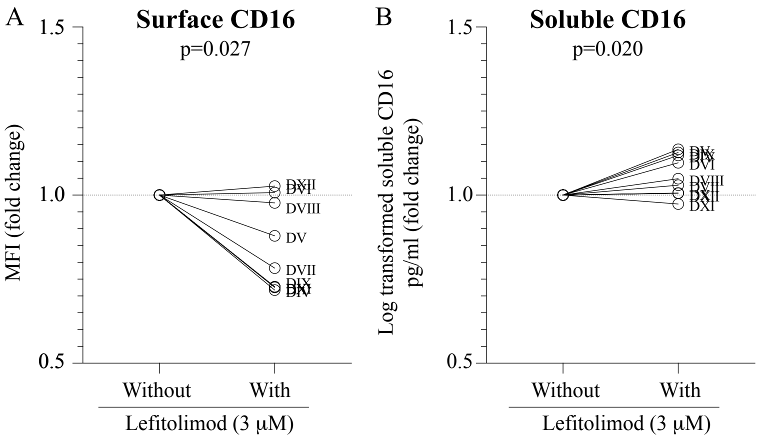

Supplemental Figure 5. Fold change data supporting Figure 4. (A-B) Fold change values highlight the differential impacts of lefitolimod on human NK cell CD16 surface levels (A) and the levels of CD16 in culture supernatants (B). Statistics: Wilcoxon test.
